# Temporal Trends in Diagnosis, Treatments, and Outcomes in Patients With Bicuspid Aortic Valve

**DOI:** 10.3389/fcvm.2021.766430

**Published:** 2021-11-04

**Authors:** Kyu Kim, Dae-Young Kim, Jiwon Seo, Iksung Cho, Geu-Ru Hong, Jong-Won Ha, Chi Young Shim

**Affiliations:** Division of Cardiology, Severance Cardiovascular Hospital, Yonsei University College of Medicine, Seoul, South Korea

**Keywords:** bicuspid aortic valve, trend, diagnosis, imaging, treatment, outcome

## Abstract

**Background:** The population is aging and advances in multimodal imaging and transcatheter valve intervention have been prominent in the past two decades. This study investigated temporal trends in demographic characteristics, use of multimodal imaging, treatments, and outcomes in patients with bicuspid aortic valve (BAV).

**Methods and Results:** A total of 1,497 patients (male 71.7%, 57 ± 14 years old) first diagnosed with BAV between January 2003 and December 2020, in a single tertiary center were divided into three groups according to year of diagnosis: group 1 (2003–2008, *n* = 269), group 2 (2009–2014, *n* = 594), and group 3 (2015–2020, *n* = 634). The patients' demographic characteristics, comorbidities, BAV morphology, BAV function, BAV-related disease, use of multimodal diagnostic imaging, treatment modality for BAV, and clinical outcomes were compared among the three groups. The ages at diagnosis and at the time of surgery/intervention increased considerably from group 1 to 3. The patients' comorbidity index also increased progressively. The proportion of non-dysfunctional BAV and significant AS increased, while that of significant AR decreased. The frequency of infective endocarditis as an initial presentation significantly decreased over time. Additionally, the use of multimodal imaging increased markedly in the most recent group. The results also indicated increasing trends in the use of bioprosthetic valves and transcatheter aortic valve replacement. Overall and cardiovascular survival rates improved from group 1 to 3 (log rank *p* < 0.001).

**Conclusions:** For the past two decades, remarkable temporal changes have occurred in patient characteristics, use of multimodal diagnostic imaging, choice of treatment modality, and clinical outcomes in patients with BAV.

## Introduction

Bicuspid aortic valve (BAV) is the most common congenital heart valve disease, and the burden of BAV disease is greater than for other congenital anomalies (1–3). BAV can present in diverse spectrum and affect valve function from non-dysfunctional to severe aortic stenosis (AS) or severe aortic regurgitation (AR) (4–6). In addition, BAV often is accompanied by aortopathy, other congenital defects, cardiomyopathies, or infective endocarditis (IE) (7–9). In the past two decades, echocardiographic surveillance of asymptomatic subjects has increased, and multimodality imaging has been developed and applied in heart valve diseases (10, 11). Recently, transcatheter aortic valve replacement (AVR) has been expanded to young age and low risk groups, and treatment methods in BAV patients have diversified (12–14). In addition, the incidence of IE as a first manifestation in BAV patients is expected to decrease as socioeconomic status improves (15). Therefore, this study aimed to investigate temporal trends in demographic characteristics, use of multimodal imaging, treatment modality, and clinical outcomes in patients with BAV from a large Korean registry.

## Methods

### Study Population

We systemically analyzed a single-center Korean registry that consisted of 1,497 consecutively enrolled BAV patients 19 years of age or older. This retrospective and prospective registry contains echocardiographic data and clinical information from medical records of the patients from January 2003 to December 2020, in Severance Cardiovascular Hospital, Seoul, Korea. All patients were diagnosed with BAV through transthoracic echocardiography, and additional diagnostic imaging was performed according to the clinical judgement of the physician. When there was discrepancy between imaging tests, we determined exclusion after comprehensive consideration of all imaging studies and intraoperative findings. There were 14 exclusions in this study. The Institutional Review Board of Severance Hospital approved this study, which was conducted in compliance with the Declaration of Helsinki. The need for informed consent was waived.

### Patient Data

Baseline characteristics at the time of diagnosis were age, sex, height, weight, body mass index, and comorbidities. The Charlson comorbidity index was calculated to determine patient risk (16). All participants in the study population underwent comprehensive transthoracic echocardiography. All echocardiographic studies were performed using commercially available equipment and were analyzed retrospectively without knowledge of the clinical data. Standard 2-dimensional and Doppler measurements were performed, and the severity of BAV dysfunction was assessed based on the American Society of Echocardiography guidelines (17, 18).

Congenital BAV was diagnosed when only two cusps were identified unequivocally in systole and diastole in the short-axis view, with a clear “fish-mouth” appearance during systole, as previously described (5–8). Type 1 was confirmed based on congenital fusion of the right and left coronary cusps; Type 2 was confirmed based on a congenital fusion of the right and non-coronary cusps; Type 3 was confirmed based on a congenital fusion of the non-coronary and left coronary cusps; Type 0 was confirmed for cases without raphe, which also is referred to as “true type” BAV. The severity of AS and AR was assessed using an integrated approach (17, 18). Patients that had at least moderate AS or moderate AR were classified as significant AS or significant AR, respectively, and others were classified with non-dysfunctional BAV (5, 6).

All measurements of the aorta were performed on the QRS complex of the electrocardiogram according to recommendations (19). The dimensions of the Valsalva sinuses were measured perpendicularly to the right and left (or non-) aortic sinuses. The sinotubular junction was measured where the aortic sinuses met the tubular aorta. The AA was measured 2 cm distal to the sinotubular junction. The presence of aortopathy was defined as an ascending aorta diameter ≥37 mm (6, 7, 20). A maximum dimension of the ascending aorta ≥45 mm was defined as severe aortopathy (6). Concomitant congenital defects including ventricular septal defect, atrial septal defect, patent foramen ovale, and patent ductus arteriosus were investigated. Concomitant cardiomyopathy was defined as specific cardiomyopathies such as hypertrophic cardiomyopathy, noncompaction cardiomyopathy, and idiopathic dilated cardiomyopathy (8). A diagnosis of IE was determined according to modified Duke criteria (21).

We investigated whether transesophageal echocardiography (TEE) and MDCT were performed in addition to transthoracic echocardiography. Diagnostic multimodal imaging was performed based on the clinician's judgement. Surgery or intervention was conducted according to the guidelines at time of diagnosis, based on patient symptoms, cardiac function, and BAV function and clinician decision. Eligibility for transcatheter AVR was determined by a multidisciplinary heart team.

The study population was divided into three groups according to year of diagnosis with six-year increments: group 1 (2003–2008, *n* = 269), group 2 (2009–2014, *n* = 594), and group 3 (2015–2020, *n* = 634). The baseline characteristics, ages at diagnosis and at the time of surgery or intervention, use of multimodality imaging, number of surgeries or interventions, and survival from all-cause death and cardiovascular death were compared among the groups.

The index date was the time of the first BAV diagnosis. Death information was collected by medical records. Cardiovascular death was defined as death due to worsening heart failure, acute coronary syndrome, cerebrovascular accidents, or sudden cardiac death. The cause of death was determined based on the death certificate.

### Statistical Analysis

Categorical variables are expressed as percentage or frequency and compared using the χ^2^ test or Fisher's exact test. Continuous variables are expressed as mean ± standard deviation. The Cochran–Armitage and Jonckheere–Terpstra methods were used to test trends in nominal and categorical variables across time periods. Survival from all-cause death and cardiovascular death was estimated using the Kaplan–Meier method and compared by Log-rank test. A probability value (*P*-value) <0.05 was considered statistically significant. Statistical analyses were conducted using R version 4.1.0 (The R Foundation for Statistical Computing; www.R-project.org).

## Results

### Temporal Trends in Characteristics of Patients With BAV

During the study period, a total of 1,497 patients (male 71.7%, 56.5 ± 14.3 years old) was diagnosed with BAV. The absolute numbers of patients diagnosed with BAV in groups 1, 2, and 3 were 269, 594, and 634, respectively. Baseline characteristics of the study population are shown in Table 1.

**Table 1:** Demographic, clinical, and imaging characteristics in the three groups.

	**Group 1** **(*n* = 269)**	**Group 2** **(*n* = 594)**	**Group 3** **(*n* = 634)**	***P* value**	***P* for trend**
Age at diagnosis, year	53.2 ± 15.1	56.7 ± 14.3	57.8 ± 13.8	<0.001	<0.001
Male sex, *n* (%)	201 (74.7)	423 (71.2)	450 (71.0)	0.290	0.290
Body mass index, kg/m^2^	23.6 ± 3.1	24.0 ± 4.0	24.2 ± 3.9	0.063	0.036
Comorbidities, *n* (%)					
Hypertension	76 (28.1)	275 (46.6)	289 (44.2)	<0.001	<0.001
Diabetes mellitus	46 (17.1)	123 (20.7)	113 (17.8)	0.314	0.862
Coronary artery disease	45 (16.7)	125 (21.0)	121 (19.1)	<0.001	0.667
Atrial fibrillation	33 (12.3)	102 (17.2)	92 (14.5)	0.148	0.732
Dyslipidemia	55 (20.4)	171 (28.8)	229 (36.1)	<0.001	<0.001
Chronic kidney disease	12 (4.5)	36 (6.1)	34 (5.4)	0.624	0.764
Liver cirrhosis	3 (1.1)	16 (2.7)	11 (1.7)	0.252	0.870
Chronic pulmonary disease	12 (4.5)	36 (6.1)	34 (5.4)	0.624	0.764
History of CVA	10 (3.7)	12 (2.0)	18 (2.8)	0.323	0.699
History of cancer	18 (6.7)	58 (9.8)	48 (7.6)	0.219	0.968
Charlson comorbidity index	1.8 ± 1.6	2.2 ± 1.8	2.2 ± 1.7	0.013	0.029
BAV morphology, *n* (%)					
Type 1, R-L fusion	161 (59.9)	364 (61.3)	374 (59.0)	0.547	0.627
Type 2, R-N fusion	40 (14.9)	96 (16.2)	113 (17.8)	0.750	0.254
Type 3, L-N fusion	14 (5.2)	27 (4.5)	24 (3.8)	0.771	0.312
Type 0, No raphe	54 (20.0)	107 (18.0)	123 (19.4)	0.716	0.969
BAV function, *n* (%)					
Non-dysfunctional AV	68 (25.3)	214 (36.0)	199 (31.4)	0.006	0.039
Significant AS	112 (41.6)	277 (46.6)	313 (49.4)	0.102	0.039
Significant AR	119 (44.2)	153 (25.8)	192 (30.3)	<0.001	0.002
Significant ASR	30 (11.2)	50 (8.4)	70 (11.0)	0.246	0.698
BAV-associated disease, *n* (%)					
Presence of aortopathy	119 (44.2)	315 (53.0)	300 (47.3)	0.030	0.913
Severe aortopathy	58 (21.6)	177 (29.8)	160 (25.2)	0.027	0.666
Coarctation of aorta	3 (1.1)	2 (0.3)	7 (1.1)	0.262	0.676
Infective endocarditis	13 (4.8)	17 (2.9)	11 (1.7)	0.027	0.010
Concomitant cardiomyopathy	10 (3.7)	17 (2.9)	24 (3.8)	0.640	0.768
Congenital defects	7 (2.6)	22 (3.7)	44 (6.9)	0.005	0.002
Multimodal imaging, *n* (%)					
TEE	60 (22.3)	210 (35.4)	245 (38.6)	<0.001	<0.001
MDCT	6 (2.2)	61 (10.3)	215 (33.9)	<0.001	<0.001
Both TEE and MDCT	4 (1.5)	42 (7.1)	126 (19.9)	<0.001	<0.001
CMR	3 (1.1)	30 (5.1)	8 (1.3)	<0.001	0.281

*AS, aortic stenosis; AR, aortic regurgitation; ASR, aortic stenosis and regurgitation; BAV, bicuspid aortic valve; TEE, transesophageal echocardiography; MDCT, multidetector computed tomography; CMR, cardiac magnetic resonance*.

**Figure 1:**
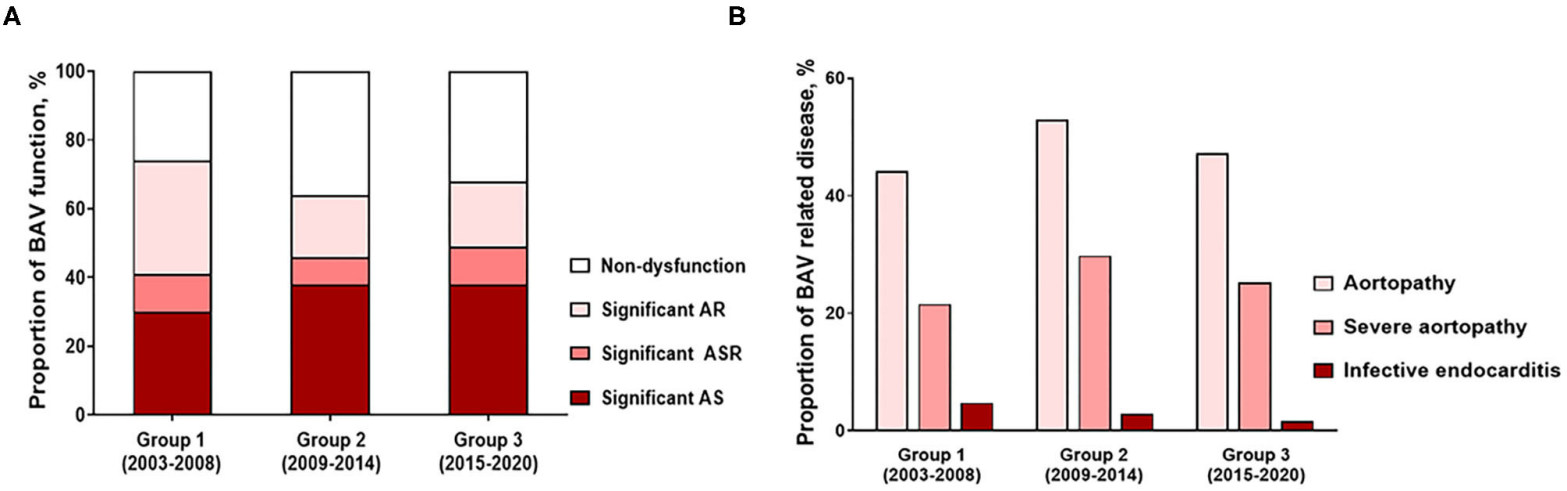
Temporal trends of **(A)** proportion of BAV function and **(B)** proportion of BAV-related disease.

**Figure 2:**
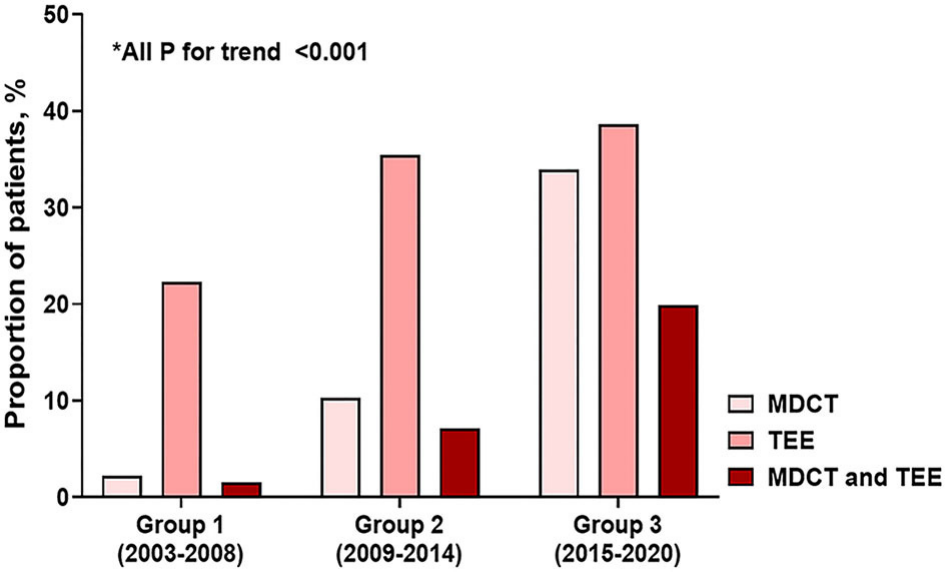
Temporal trends in the use of additional diagnostic imaging.

Patient demographics indicated that age at diagnosis increased significantly from group 1 to group 3 (*P* for trend < 0.001), and sex distribution was not significantly different between groups according to diagnosis year, with males accounting for more than 70% of all groups. From group 1 to 3, a tendency for an increase in body mass index was observed (*P* for trend = 0.036). Analysis of patient comorbidities indicated that the more recent patients experienced more frequent hypertension and dyslipidemia, and the Charlson comorbidity index increased in this group (*P* for trend = 0.029). Analysis of BAV morphology indicated that type 1 was dominant in all groups, and there was no difference according to group. In terms of BAV function, the diagnosis of non-dysfunctional BAV increased in groups 2 and 3 compared to group 1 (*P* for trend=0.039), the proportion of significant AR decreased (*P* for trend = 0.002), and the significant AS increased steadily (*P* for trend = 0.039; Figure 1A). In terms of BAV-associated disease, about half of the patients had aortopathy, and about one-quarter had severe aortopathy, with no significant trends observed over time (Figure 1B). From group 1 to 3, the prevalence of infective endocarditis significantly decreased (*P* for trend = 0.010; Figure 1B). Detection of congenital defects increased (*P* for trend = 0.002) and likely was attributable to the increased use in additional diagnostic imaging from groups 1 to 3. In group 3, TEE and MDCT were used in 38.6 and 33.9% of patients, respectively (Figure 2).

### Temporal Trends for Treating BAV-Related AV Disease

**Figure 3:**
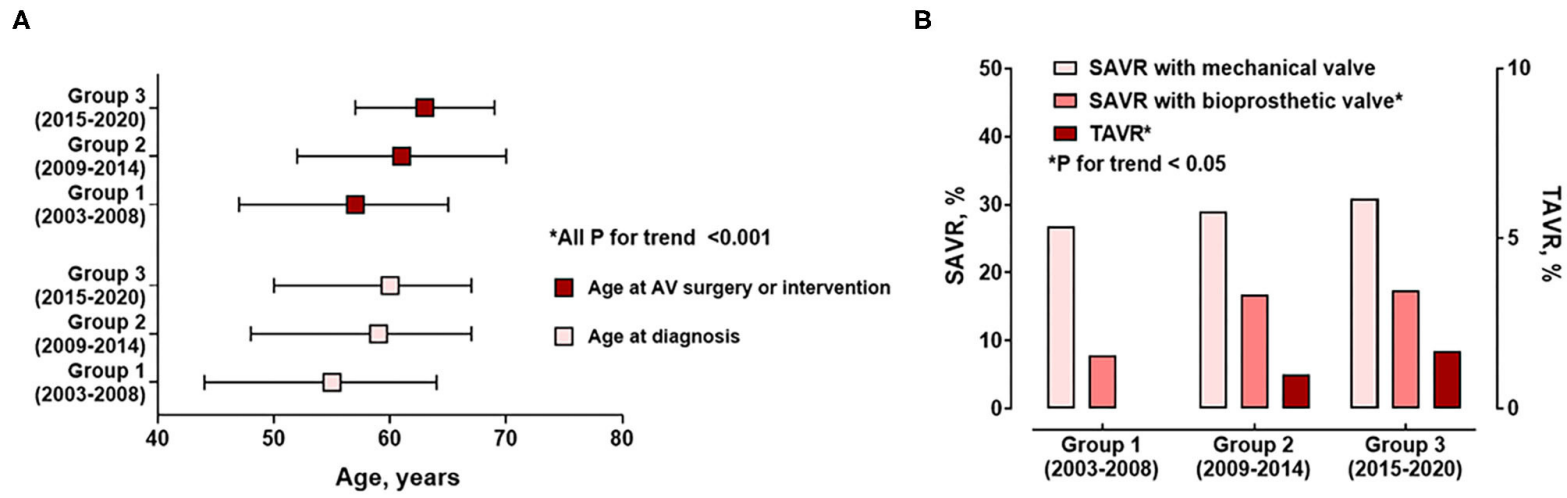
Temporal trends of **(A)** ages at diagnosis and AV surgery or interventions and **(B)** treatment methods.

Table 2 shows treatment characteristics for the three groups of the study population. There was an increasing trend in age at surgery or intervention between groups (*P* for trend < 0.001; Figure 3A). The mean age at surgery or intervention in group 1 was 55 years, while that in group 3 was 62 years. The proportion of patients older than 70 years at surgery or intervention remarkably increased (*P* for trend = 0.003) and reached about 25% in groups 2 and 3. In terms of indications for surgery or intervention, surgery due to severe AR or infective endocarditis decreased over time (*P* for trend = 0.024, 0.027, respectively).

**Table 2:** Treatment approaches in the three groups.

	**Group 1** **(*n* = 269)**	**Group 2** **(*n* = 594)**	**Group 3** **(*n* = 634)**	***P* value**	***P* for trend**
Surgery/intervention, *n* (%)	123 (45.7)	281 (47.3)	315 (49.7)	0.498	0.309
Age at surgery/intervention, years	54.5 ± 12.8	59.7 ± 12.7	61.7 ± 12.0	<0.001	<0.001
<30, *n* (%)	6 (2.2)	5 (0.8)	5 (0.8)	0.123	0.094
30–49, *n* (%)	30 (11.2)	47 (7.9)	37 (5.8)	0.021	0.006
50–69, *n* (%)	75 (27.9)	156 (26.3)	195 (30.8)	0.213	0.218
≥70, *n* (%)	12 (4.5)	73 (12.3)	78 (12.3)	0.001	0.003
Indications for surgery/intervention					
Severe AS, *n* (%)	97 (36.1)	204 (34.3)	232 (36.6)	0.702	0.731
Severe AR, *n* (%)	64 (23.8)	92 (15.5)	103 (16.2)	0.008	0.024
Severe ASR, *n* (%)	13 (4.8)	4 (1.3)	17 (2.7)	0.010	0.311
Severe aortopathy, *n* (%)	58 (21.6)	177 (29.8)	169 (25.2)	0.027	0.666
Infective endocarditis, *n* (%)	10 (3.7)	16 (2.7)	9 (1.4)	0.086	0.027
Surgical AVR or repair, *n* (%)	119 (44.2)	271 (45.6)	298 (47.0)	0.731	0.448
Bioprosthetic valve	28 (10.4)	96 (16.2)	99 (15.6)	0.071	0.105
Mechanical valve	89 (33.1)	175 (29.5)	199 (31.4)	0.536	0.809
Aortic valve repair	2 (0.7)	3 (0.5)	3 (0.5)	0.871	0.646
Surgery for aorta, *n* (%)	27 (10.0)	98 (16.5)	112 (17.7)	0.012	0.009
Isolated aorta surgery	4 (1.5)	4 (0.7)	6 (0.9)	0.516	0.605
Concomitantly with AV surgery	23 (8.6)	94 (15.8)	106 (16.7)	0.005	0.003
Concomitant surgery, *n* (%)	2 (0.7)	18 (3.0)	68 (10.7)	<0.001	<0.001
Coronary artery bypass	2 (0.7)	8 (1.3)	19 (3.0)	0.032	0.012
Other surgery^*^	0 (0.0)	10 (1.7)	52 (8.2)	<0.001	<0.001
Transcatheter AVR, *n* (%)	0 (0.0)	6 (1.0)	11 (1.7)	0.074	0.024

*AS, aortic stenosis; AR, aortic regurgitation; ASR, aortic stenosis and regurgitation; AVR, aortic valve replacement; AV, aortic valve*.

* *Other surgery included patch repair of ventricular or atrial septal defect, direct closure of patent foramen ovale, and patent ductus arteriosus ligation*.

**Figure 4:**
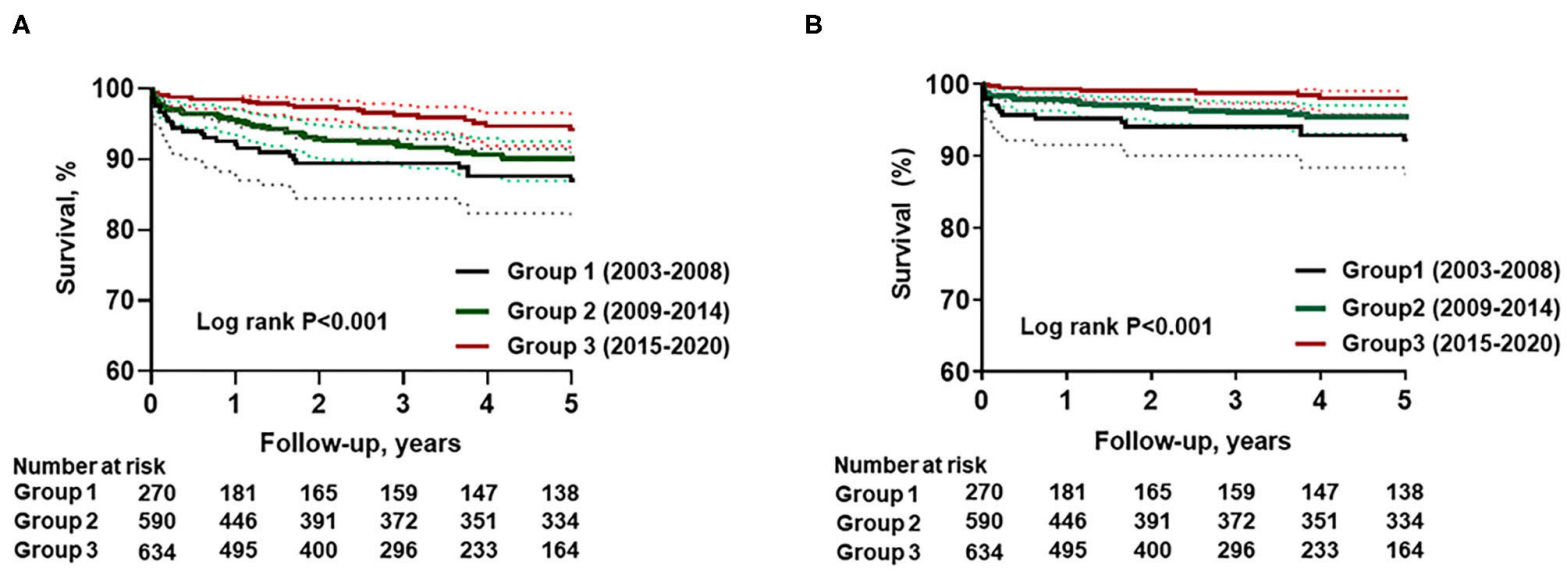
Temporal trends in clinical outcomes: **(A)** survival from all-cause death, **(B)** survival from cardiovascular death.

As age at surgery increased from group 1 to 3, surgical AVR using bioprosthetic valves significantly increased (*P* for trend = 0.002). In addition, transcatheter AVR gradually increased over time (*P* for trend = 0.024; Figure 3B). The results indicate that there were more frequent concomitant surgeries such as coronary artery bypass for patients diagnosed and treated more recently (*P* for trend = 0.002). During the 3.8 years (interquartile range 1.0–6.9 years) of follow-up, all-cause death and cardiovascular death significantly decreased from group 1 to group 3 (both log-rank *P* < 0.001; Figure 4).

## Discussion

The principal findings of this study were as follow: (1) a significant temporal increase was observed in both age at the time of diagnosis and age at the time of surgery or intervention; (2) over time, the proportions of non-dysfunctional BAV and significant AS increased and significant AR decreased in patients with BAV; (3) a temporal change in the incidence of infective endocarditis was observed in patients with BAV; (4) surgical AVR using bioprosthetic valve and transcatheter AVR increased; (5) the frequency of additional diagnostic imaging, such as TEE or MDCT, remarkably increased in patients with BAV; and (6) there was a recent significant improvement in all-cause and cardiovascular death among all patients diagnosed with BAV. Understanding these temporal changes and trends in patient characteristics, BAV function, diagnosis, treatment, and outcome will be important for further diagnostic and treatment advances.

### Temporal Trends in Patient Characteristics, Diagnosis, and Treatment

BAV is the most common adult congenital heart defect and has associated increased risk for severe AS or AR, thoracic aortic disease or acquired complications such as IE (1–4, 22, 23). Because BAV is congenital, BAV-related diseases typically manifest at an early age. Therefore, the majority of previous studies has reported a mean age of about 40 years (24). Significant bicuspid AS usually occurs earlier than tricuspid AS and is reported in their 50s and 60s (25). In patient with BAV, AR is more common at a young age, whereas AS usually presents later in life (26). In this study, the mean age at diagnosis was 56.5 years, and the mean age increased over about two decades. Furthermore, the mean age at surgery or intervention was 61.6 years, and 24.5% of patients in the more recent group were older than 70 years. In addition, as the global burden of calcified aortic valve disease increased, the proportion of significant AR decreased while that of significant AS increased from group 1 to 3, likely related to the increase in age for the general population (27). As ages of patients at diagnosis and at surgery or intervention increased, the treatment strategy for BAV dysfunction also changed. In this study, 35.6% of patients in group 3 underwent AVR with bioprosthetic valves. Increased use of bioprosthetic valves was an expected finding because of the increasing aging trend in patient with BAV. In addition, as the comorbidities of patients increased, the surgical risk also increased, as did the demand for transcatheter AVR. This study showed increasing trend of transcatheter AVR in BAV patients after its launch in 2011, in Korea.

The diagnosis of non-dysfunctional BAV had increased in this study. In Korea, the number of TTE as screening tool is continuously increasing (28). The increased number of exam might enable early diagnosis of BAV and related disease in general population.

The present study also showed a decreasing trend for prevalence of IE as the first manifestation of BAV disease. The rate of IE was 1.7% in group 3, and the incidence of IE in BAV has been reported around 2%, which was comparable to our results (9, 24). A recent report from the United States showed decreasing trend of native valve endocarditis but that of increased prosthetic valve and device-related endocarditis (15). These trends might be derived from increased echocardiographic surveillance for BAV and improved socioeconomic status over time.

### Temporal Trends for Multimodal Imaging in BAV Patients

Multimodality imaging has become increasingly important because BAV is not only a valve disease, but also is associated with other diseases such as aortopathy and cardiomyopathies (6–8, 10). The first diagnostic tool of choice to evaluate heart valves was transthoracic echocardiography because it is easy to use and noninvasive. Recently, MDCT has been used as a complement to echocardiography for diagnosing heart valve disease and preoperative evaluation (18, 29, 30). In patients with BAV, MDCT can provide accurate information about the BAV and adjacent structural abnormalities including the aorta, concomitant anomalies, or combined coronary artery disease (10, 11). Furthermore, in the era of transcatheter AVR, the use of multimodal imaging is becoming an increasingly essential part of routine clinical practice, particularly for BAV patients with significant AS (10, 31). The patients with BAV had chance of concomitant cardiomyopathies. They had different flow dynamics from the patients with tricuspid aortic valve (32). Furthermore, myocardial fibrosis has been reported as important prognostic factor in BAV related disease such as AS or AR (33, 34). Cardiac magnetic resonance might be useful in patients with BAV and related disease (8, 35). The results of this study indicate that the use of multimodal imaging has increased, and that this approach can detect concomitant disease such as congenital defects based on the overall trends in diagnostic imaging in patients with BAV.

### Temporal Trends of Clinical Outcomes in BAV Patients

This study also showed clinical outcome improvements in the more recent group despite an increase in mean age with a higher comorbidity index. There are several factors that could impact these results. Notably, as the proportion of non-dysfunctional BAV increased in group 3, it is possible that fewer clinical events were diagnosed because those events likely were attributable to previously undetected non-dysfunctional BAV in patients. In addition, recent advances in diagnostic imaging, surgical techniques (36), medical systems such as a multidisciplinary approach, and application of transcatheter AVR in patients with high surgical risk might influence the improved clinical outcomes in patients with BAVs.

### Study Limitations

Our study had several limitations. First, this study was conducted at a single tertiary center by comprehensively reviewing retrospective and prospective data; therefore, selection and referral bias were inevitable, and our results could not be generalized. The proportions of significant BAV dysfunction and severe aortopathy were higher than reported in previous studies (24, 26). The clinical follow-up duration for the study population was relatively short. Seconds, the study subjects were diagnosed based on TTE, so there might be inevitable limitations and bias for the morphologic evaluation of BAV, particularly severely calcified aortic valve. However, as this study had additional imaging studies performed by clinician's judgement and consisted of a large-scale population, we believe this potential bias would not change our main findings. Additionally, it is difficult to generalize and apply these temporal trends to other societies or countries because if differing social and medical environmental factors. Despite these limitations, we believe that the data from this large Korean registry will be helpful to understand the characteristics, diagnosis, treatment, and outcomes for BAV patients over the past two decades. Further, some of the temporal trends might be applicable to other societies.

## Conclusions

In past two decades, there have been remarkable temporal changes in patients with BAV. Patient characteristics, proportion of BAV dysfunction, diagnosis, and treatment strategy have changed, and the demand for bioprosthetic valves has increased. Temporal trends were observed with improvements of clinical outcomes in patients with BAV.

## Data Availability Statement

The raw data supporting the conclusions of this article will be made available by the authors, without undue reservation.

## Ethics Statement

The studies involving human participants were reviewed and approved by Severance Hospital, Yonsei University College of Medicine. Written informed consent for participation was not required for this study in accordance with the national legislation and the institutional requirements.

## Author Contributions

KK and CS contributed to the conception, design of the work, and drafted the manuscript. D-YK, JS, and IC assisted in data collection and analysis. G-RH and J-WH contributed to the review and revision of the manuscript. All authors contributed to the article and approved the submitted version.

## Funding

This research was supported from the Korean Cardiac Research Foundation (202103-02).

## Conflict of Interest

The authors declare that the research was conducted in the absence of any commercial or financial relationships that could be construed as a potential conflict of interest.

## Publisher's Note

All claims expressed in this article are solely those of the authors and do not necessarily represent those of their affiliated organizations, or those of the publisher, the editors and the reviewers. Any product that may be evaluated in this article, or claim that may be made by its manufacturer, is not guaranteed or endorsed by the publisher.

## Abbreviations

AR: aortic regurgitation
AS: aortic stenosis
AVR: aortic valve replacement
BAV: bicuspid aortic valve
IE: infective endocarditis
MDCT: multidetector computed tomography
TEE: transesophageal echocardiography.
